# Supplementary material for: BCL-XL directly modulates RAS signalling to favour cancer cell stemness
Source: Nat Commun. 2017 Oct 24;8:1123. doi: 10.1038/s41467-017-01079-1 (PMC5654832; doi:10.1038/s41467-017-01079-1)
Supplement: Supplementary file 1 — Supplementary Information [file 41467_2017_1079_MOESM1_ESM.pdf]

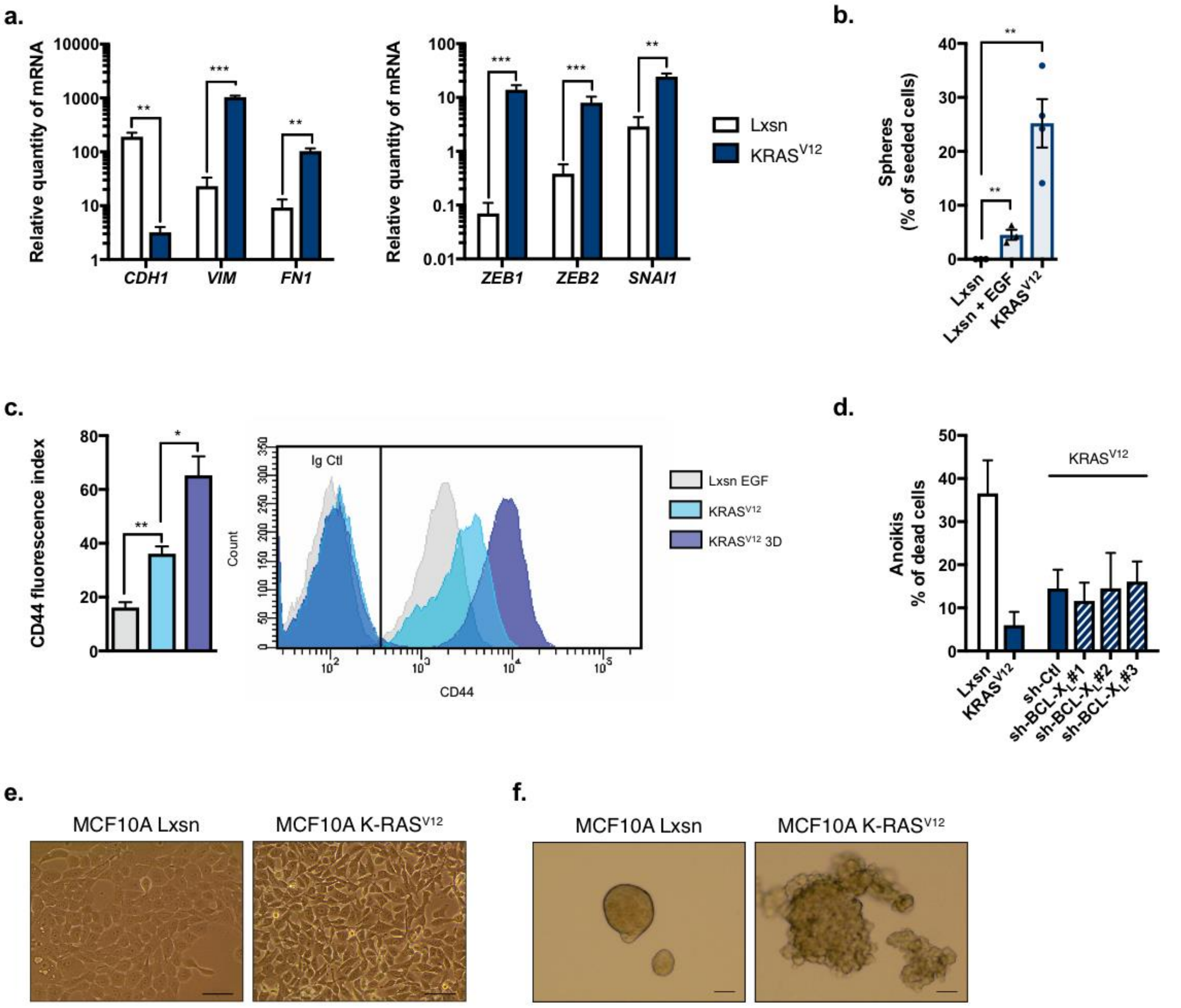

Supplementary Figure 1 | Characterization of KRAS^{V12}-transformed MCF10A cells

a. qPCR of EMT-related mRNA expressed in MCF10A Lxsn and KRAS^{V12} cell lines grown in adherent conditions. Mean and SEM of 3 independent experiments are represented as relative quantity of mRNA normalised to the mean of *RPLP0*, *RPS18* and *B2M* relative expression (two-tailed unpaired t-test) b. Percentage of sphere forming cells in bulk population of EGF-treated MCF10A Lxsn cells or MCF10A KRAS^{V12} cells. Mean of 4 independent experiments are represented with SEM (two-tailed unpaired *t*-test) c. Flow cytometry analysis of CD44 expression on EGF-treated MCF10A Lxsn and MCF10A KRAS^{V12} cell lines in adherent condition and on KRAS^{V12}-induced spheres. Adherent cells were harvested using trypsin-EDTA and spheres were collected after a week growing in serum-free media in ultra-low adhesion plates and then dissociated with trypsin-EDTA before staining. One representative experiment is represented on the left and the mean and SEM of 7 (EGF-treated and KRAS^{V12} cells) or 4 (KRAS^{V12}-induced spheres) independent experiments are represented on the right (two-tailed paired *t*-test) d. Anoikis of MCF10A EGF-treated Lxsn and KRAS^{V12} priory infected or not with the indicated sh-RNA during 72h was estimated by eosin assay. Cells were cultivated in DMEM-F12 5% DHS with 0.5% of methylcellulose during 48h. Cells were then collected, stained with eosin and percentage of red cells representing the percentage of dead cells was calculated. Mean and SEM of 3 independent experiments are represented e. Phase-contrast microscopic images of MCF10A Lxsn and MCF10A KRAS^{V12} in adherent condition (magnitude 20X, bar: 20µm) f. Phase-contrast microscopic images of MCF10A Lxsn and MCF10A KRAS^{V12} in mammosphere condition (magnitude 10X, bar: 300µm)

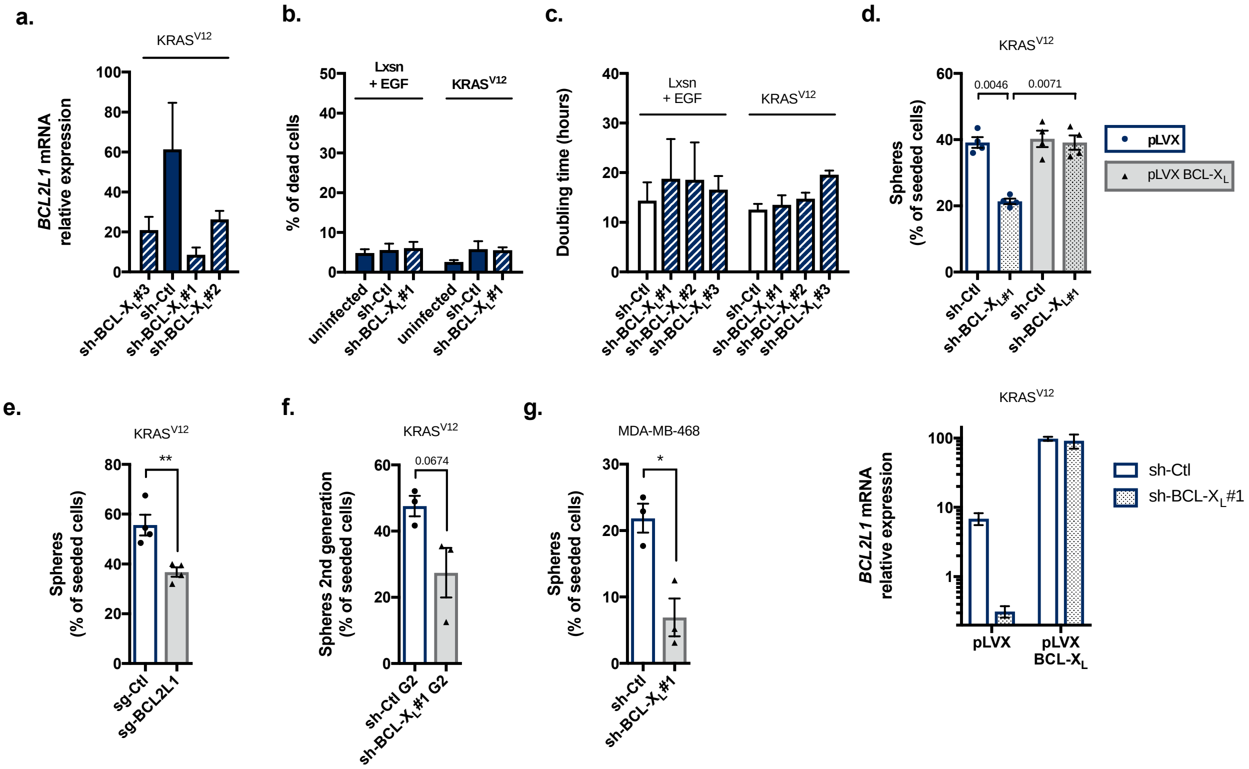

Supplementary Figure 2 | BCL-X_L knockdown does not affect survival or proliferation of RAS-transformed cells but affects second generation sphere forming

a. qPCR of *BCL2L1* mRNA in MCF10A and KRAS^{V12} cell line infected with the indicated sh-BCL-X_L during 72h. Mean and SEM of 3 independent experiments are represented as relative quantity of mRNA normalised to the mean of *RPLP0*, *RPS18* and *B2M* relative expression b. Cell death of MCF10A EGF-treated Lxsn and KRAS^{V12} infected or not with sh-BCL-X_L for 72h was estimated by trypan blue assay. Mean and SEM of 3 (uninfected conditions) or 6 (infected conditions) independent experiments are represented c. Doubling time of MCF10A KRAS^{V12} EGF-treated cells and MCF10A KRAS^{V12} cells after infection with the indicated sh-RNA for 72h. Mean and SEM of 3 independent experiments are represented d. Up: Percentage of sphere forming cells from KRAS^{v12} cell line expressing a sh-BCL-X_L #1 resistant BCL-X_L (the corresponding lentivector was named pLVX BCL-X_L for the sake of simplicity) or not (pLVX) 72h after infection with the indicated lentivector sh-RNAs. 128 cells per condition were seeded in serum-free media in ultra-low adhesion plates and the resulting spheres counted after two weeks incubation. Each dot represents the percentage of cell forming sphere for one biological replicate. Mean and SEM of 3 independent experiments are represented (two-tailed unpaired t-test). Bottom: relative quantity of *BCL2L1* mRNA normalised to the mean of *RPLP0*, *RPS18* and *ACTB* relative expression by qPCR for each condition used on the left. e. Percentage of sphere forming cells from KRAS^{v12} knock out for BCL-X_L(sg-*BCL2L1*) or KRAS^{v12} control (sg-Ctl) (see Supplementary Figure 5a for western blot immunodetection of BCL-X_L) f. Percentage of sphere forming cells from a population of first generation spheres in MCF10A KRAS^{V12} cells priory infected cells with sh-*BCL2L1*. Mean and SEM of 3 independent experiments are represented (two-tailed unpaired *t*-test) g. Percentage of sphere forming cells in bulk populations of MDA-MB-468 cells estimated after infection with the indicated sh-BCL-X_L for 72h. Mean and SEM of 3 independent experiments are represented (two-tailed unpaired t-test)

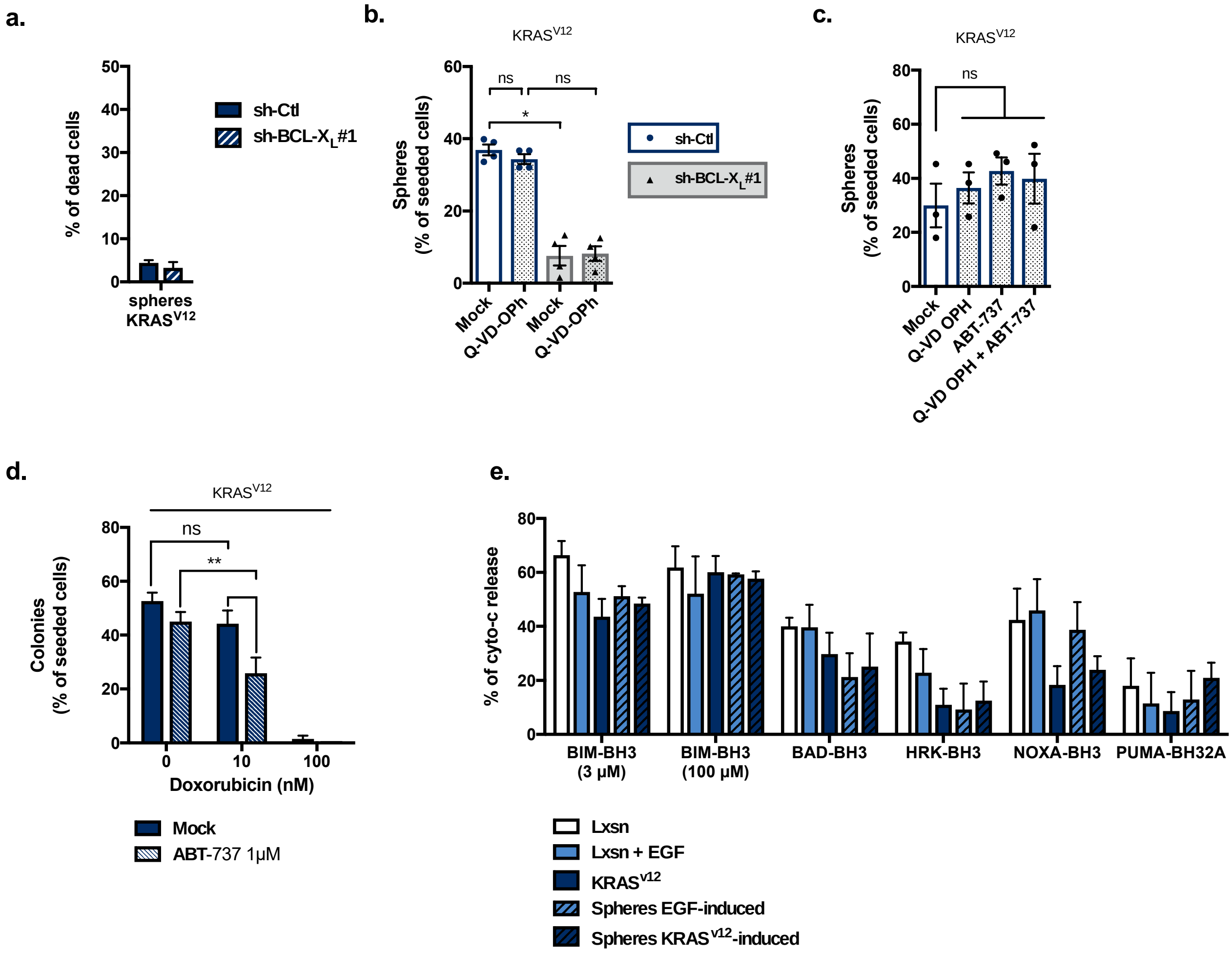

Supplementary Figure 3 | BCL-X_L impact on CIC maintenance does not involve its anti-apoptotic function

a. Cell death in spheres from MCF10A KRAS^{V12} cells infected with sh-*BCL2L1* or a control vector was estimated by trypan blue assay. Cells were infected during 72h then seeded in serum-free media in ultra-low adhesion plates for a week. Spheres were then dissociated with Trypsin-EDTA and stained with trypan blue. Mean and SEM of 3 independent experiments are represented b. Cell forming sphere from MCF10A KRAS^{V12} cells infected with sh-BCL-X_L or a control vector and treated with 10 µM Q-VD-OPh. Cells were infected during 72h then seeded in serum-free media in ultra-low adhesion plates for a week with or without Q-VD-OPh. 128 cells per condition were seeded and each dot represents the percentage of cell forming sphere for one biological replicate. Mean and SEM of 4 independent experiments are represented c. Cell forming sphere from MCF10A KRAS^{V12} cells treated with 10 µM Q-VD-OPh and/or 1 µM ABT-737. Cells were pre-treated during 72h then seeded in serum-free media in ultra-low adhesion plates for a week with 10 µM Q-VD-OPh and/or 1 µM ABT-737. 128 cells per condition were seeded and each dot represents the percentage of cell forming sphere for one biological replicate. Mean and SEM of 3 independent experiments are represented d. Percentage of cell forming colony in MCF10A KRAS^{V12} cell line treated with 1 µM ABT-737 and/or different doses of Doxorubicin. Cells were seeded at low density with drugs and colonies were counted after a week of incubation period. Mean and SEM of 7 independent experiments are represented (two-tailed paired *t*-test) e. BH3-profiling of the indicated cells treated with the indicated peptide was performed as described in Methods. Results expressed as % of cyto-c release in treated cells compare to control cells

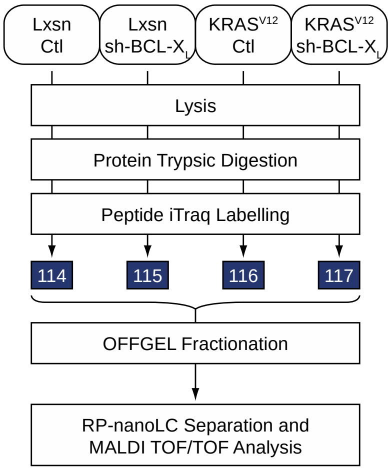

Supplementary Figure 4 | Illustration of the iTRAQ quantitative proteomic approach MALDI TOF/TOF analysis workflow

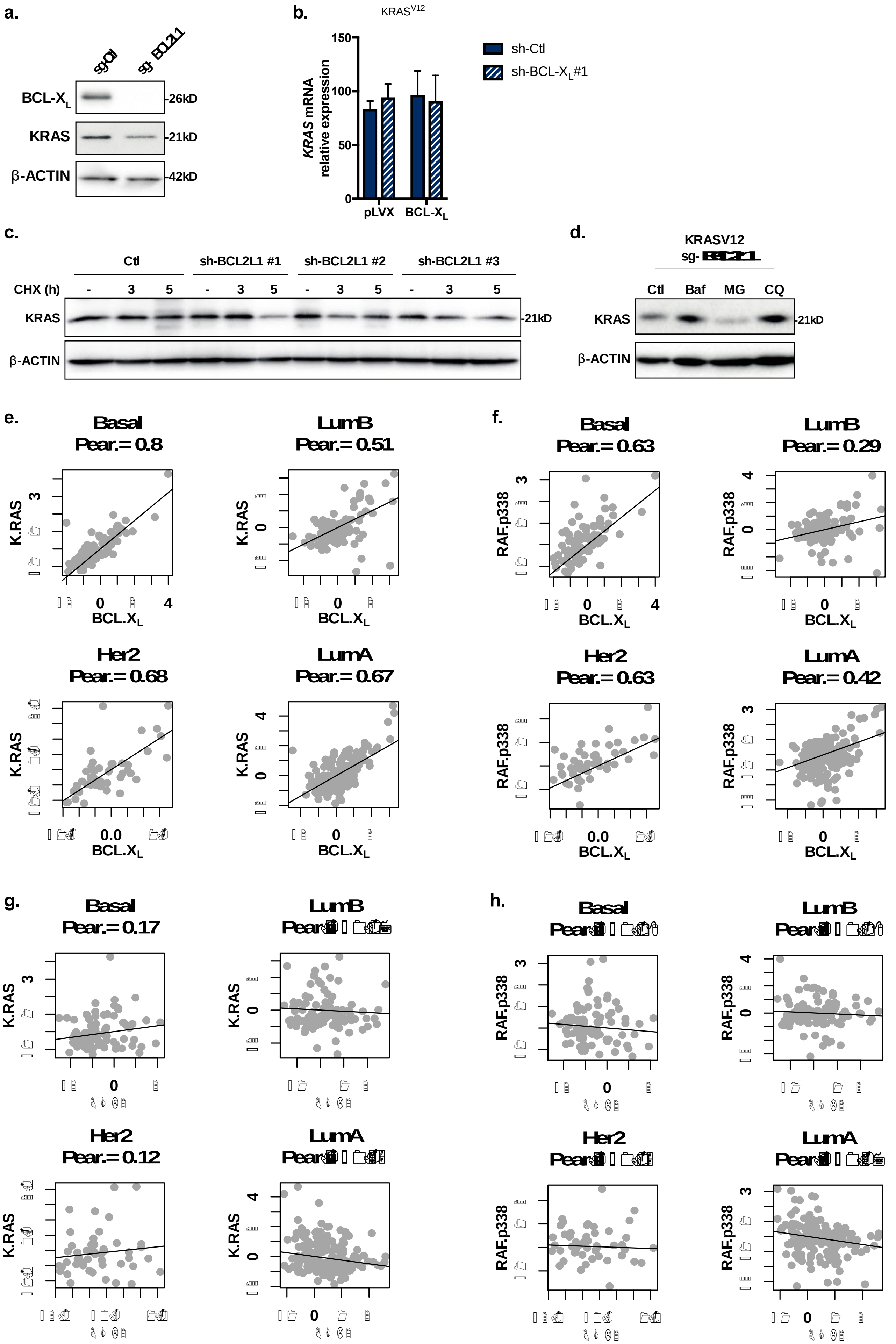
a.
sgCtl
sg-BCL2L1
BCL-XL
KRAS
β-ACTIN
26kD
21kD
42kD
b.
KRASV12
KRAS mRNA relative expression
150
100
50
0
pLVX
BCL-XL
sh-Ctl
sh-BCL-XL#1
c.
Ctl
sh-BCL2L1 #1
sh-BCL2L1 #2
sh-BCL2L1 #3
CHX (h)
- 3 5
KRAS
β-ACTIN
21kD
d.
KRASV12
sg-
Ctl
Baf
MG
CQ
KRAS
β-ACTIN
21kD
e.
Basal Pear.= 0.8
LumB Pear.= 0.51
Her2 Pear.= 0.68
LumA Pear.= 0.67
K.RAS
BCL.XL
f.
Basal Pear.= 0.63
LumB Pear.= 0.29
Her2 Pear.= 0.63
LumA Pear.= 0.42
RAF.p338
BCL.XL
g.
Basal Pear.= 0.17
LumB
Her2 Pear.= 0.12
LumA
K.RAS
h.
Basal
LumB
Her2
LumA
RAF.p338

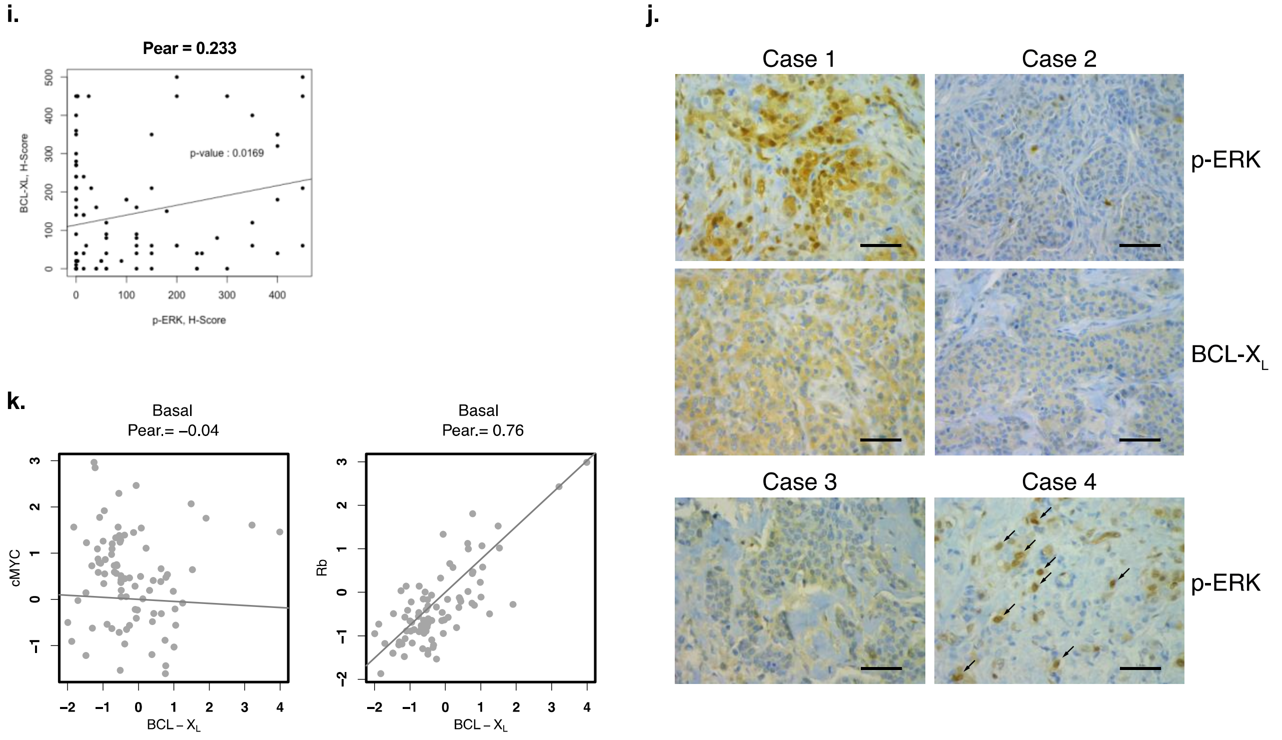

Supplementary Figure 5 | BCL-X_L favours KRAS protein expression *in vitro* and its expression is correlated with KRAS expression and p-RAF (S338) in TNBC

a. Western blot analysis of BCL-X_L expression in $KRAS^{V12}$ cell line knocked out for *BCL2L1 (sg-BCL2L1)* or in $KRAS^{V12}$ control cell line *(sg-Ctl)* b. Mean and SEM of 3 independent experiments are represented as relative quantity of *KRAS* mRNA normalised to the mean of *RPLP0*, *RPS18* and *B2M* relative expression in $KRAS^{v12}$ cell line expressing a sh-BCL-X_L #1 resistant BCL-X_L (the corresponding lentivector was named BCL-X_L for the sake of simplicity) or not (pLVX) 72h after infection with the indicated lentivector sh-RNAs c. Western blot analysis of KRAS expression in MCF10A $KRAS^{V12}$ cells infected with three different sh-RNAs against BCL-X_L for 72h then treated with 5 µg.ml^{-1} of a protein synthesis inhibitor, Cycloheximide (CHX) for 3 or 5h. β-ACTIN expression was used as loading control d. Western blot analysis of KRAS expression in MCF10A $KRAS^{V12}$ cells knocked out for *BCL2L1.* Cells were incubated 24h at 37 °C with 10 nM Bafilomycin, 3 µM MG132 or 50 µM chloroquin as indicated, then proteins were extracted and immunoblotted e, f, g, h. Correlations between KRAS protein expression or p338RAF protein expression and BCL-X_L (c, d) or BCL-2 protein expression (e, f) in distinct breast cancer subtypes (Luminal A, Luminal B, HER2, and Basal as indicated). The results shown are based upon data generated by the TCGA Research Network: http://cancergenome.nih.gov/ (see Methods for more details). Quantified expressions from RPPA data were examined for correlation using Pearson's (Pear.) analysis i. Representation of p-ERK and BCL-X_L H-scores in TNBC. Correlation is evaluated using the "linear model" function in R j. Expression of p-ERK and BCL-XL in primary TNBC.Top; IHC staining showing examples of intense (Case 1) and weak (Case 2) staining of p-ERK and BCL-X_L proteins in two representative cases (400×, Scale bars: 10 µm). Bottom; IHC staining showing examples of cytoplasmic (Case 3) and nuclear (Case 4) staining of p-ERK proteins in two different cases. Arrows show the cells where p-ERK is nuclear (400×, Scale bars: 10 µm) k. Correlations between BCL-X_L and cMYC or Rb expression in basal subtype tumour samples were investigated as described above in e-h

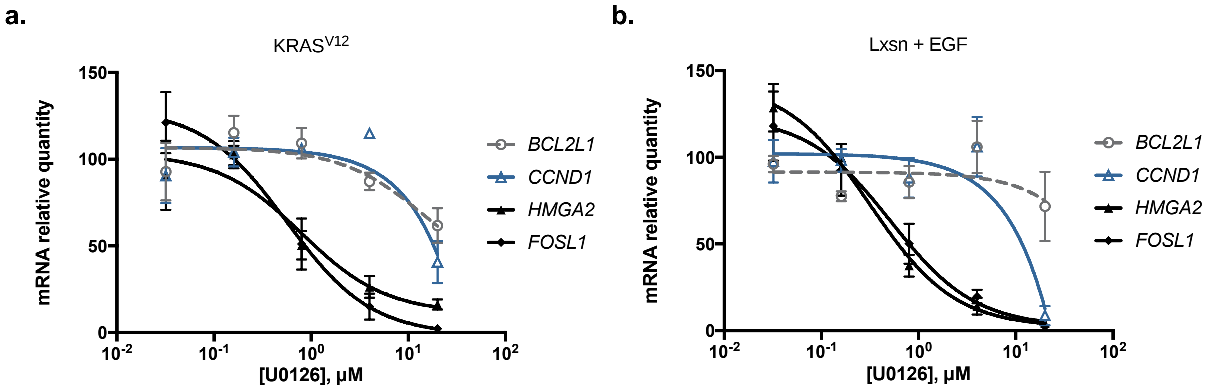

Supplementary Figure 6 | BCL-X_L-dependent RAS target genes expression are sensitive to low dose of MEK inhibitor

a. qPCR analysis of *BCL2L1*, *HMGA2*, *FOSL1*, *BCL2L11* and *CCND1* mRNA in MCF10A KRAS^{V12} cells treated with increasing doses of MEK inhibitor (U0126) during 24 hours. Mean and SEM of 3 independent experiments are represented as relative quantity of mRNA normalised to the mean of *RPLP0*, *RPS18* and *ACTB* relative expression. b. qPCR of *BCL2L1*, *HMGA2*, *FOSL1*, *BCL2L11* and *CCND1* mRNA in MCF10A Lxsn cells treated with increasing doses of U0126 during 24 hours in presence of 20 ng.ml^{-1} EGF. Mean and SEM of 3 independent experiments are represented as relative quantity of mRNA normalised to the mean of *RPLP0*, *RPS18* and *GAPDH* relative expression.

a.

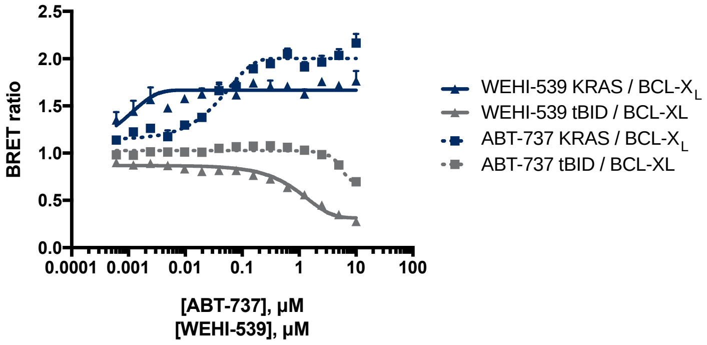

Supplementary Figure 7 | The BH3 domain of BCL-X_L is not involved in is interaction with KRAS

Sensitivity of interactions between RLuc-KRAS or RLuc-tBID and YFP-BCL-X_L to WEHI-539 or ABT-737 treatments were assessed using BRET interaction assays on a fixed donor (RLuc-fused proteins)/acceptor (YFP-fused protein) ratio treated with increased concentrations of BH3 mimetics in MCF-7 cells

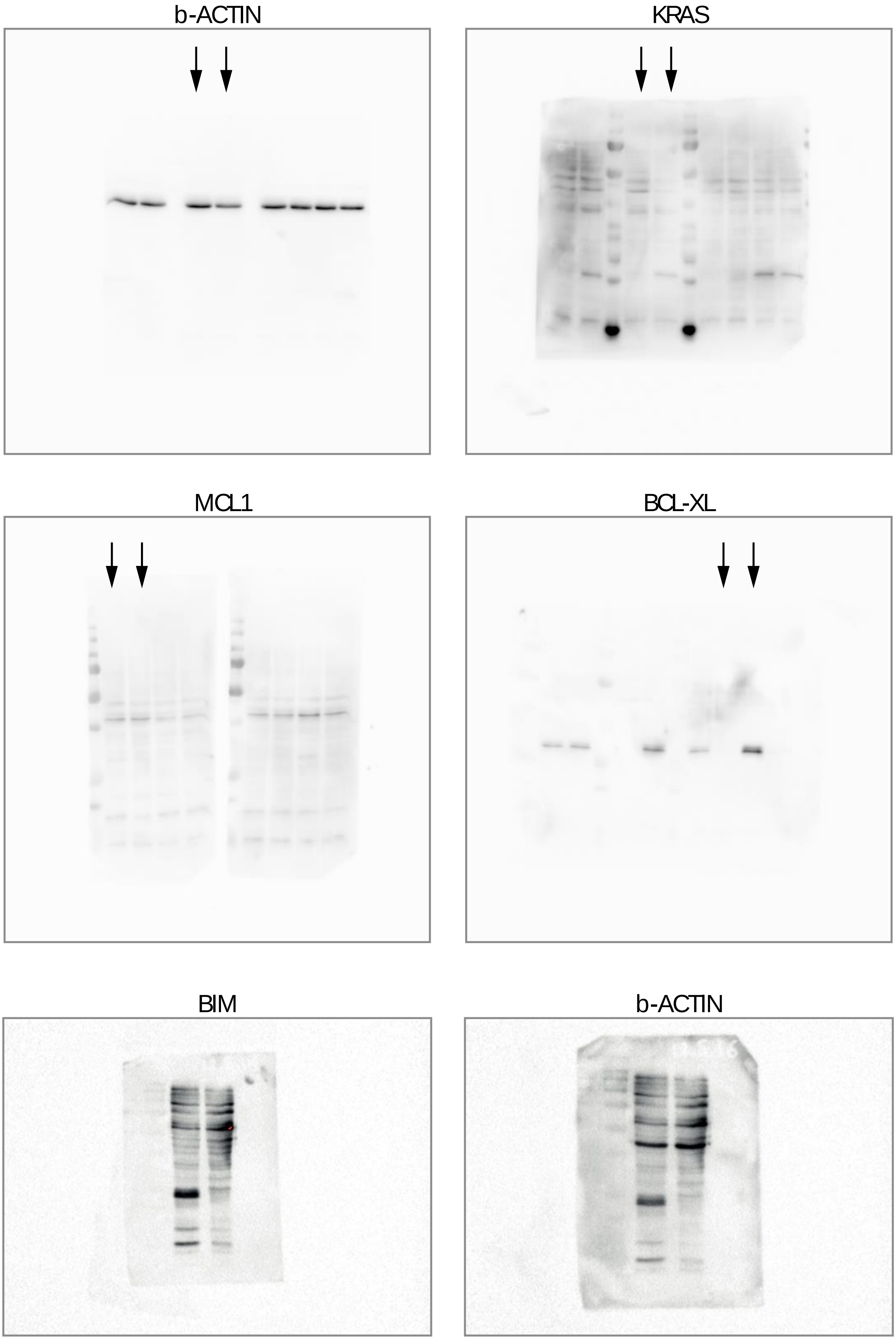

Supplementary Figure 8 | Full blots corresponding to portion of blots presented in Figure 1c
The lanes used in this study are indicated with arrows

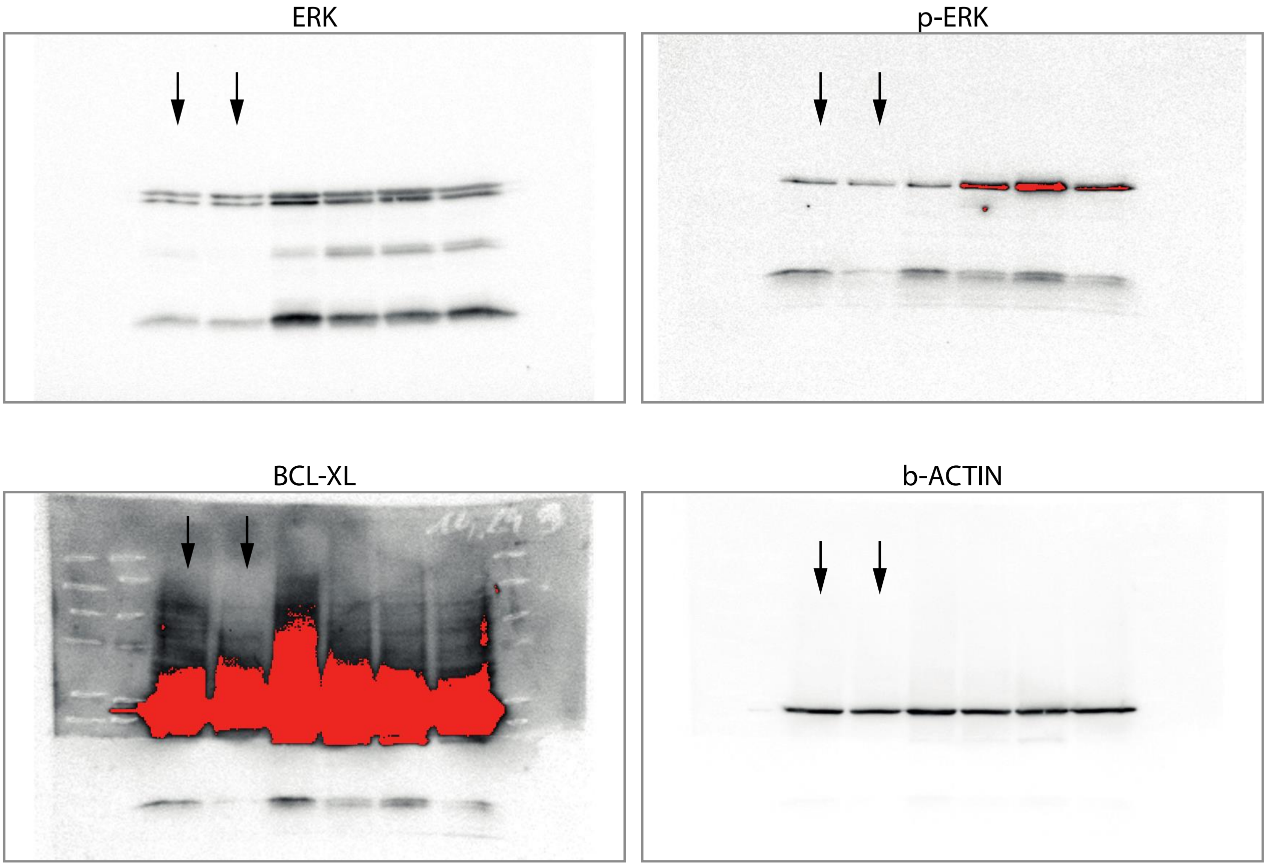

Supplementary Figure 8 | Full blots corresponding to portion of blots presented in Figure 2b
The lanes used in this study are indicated with arrows

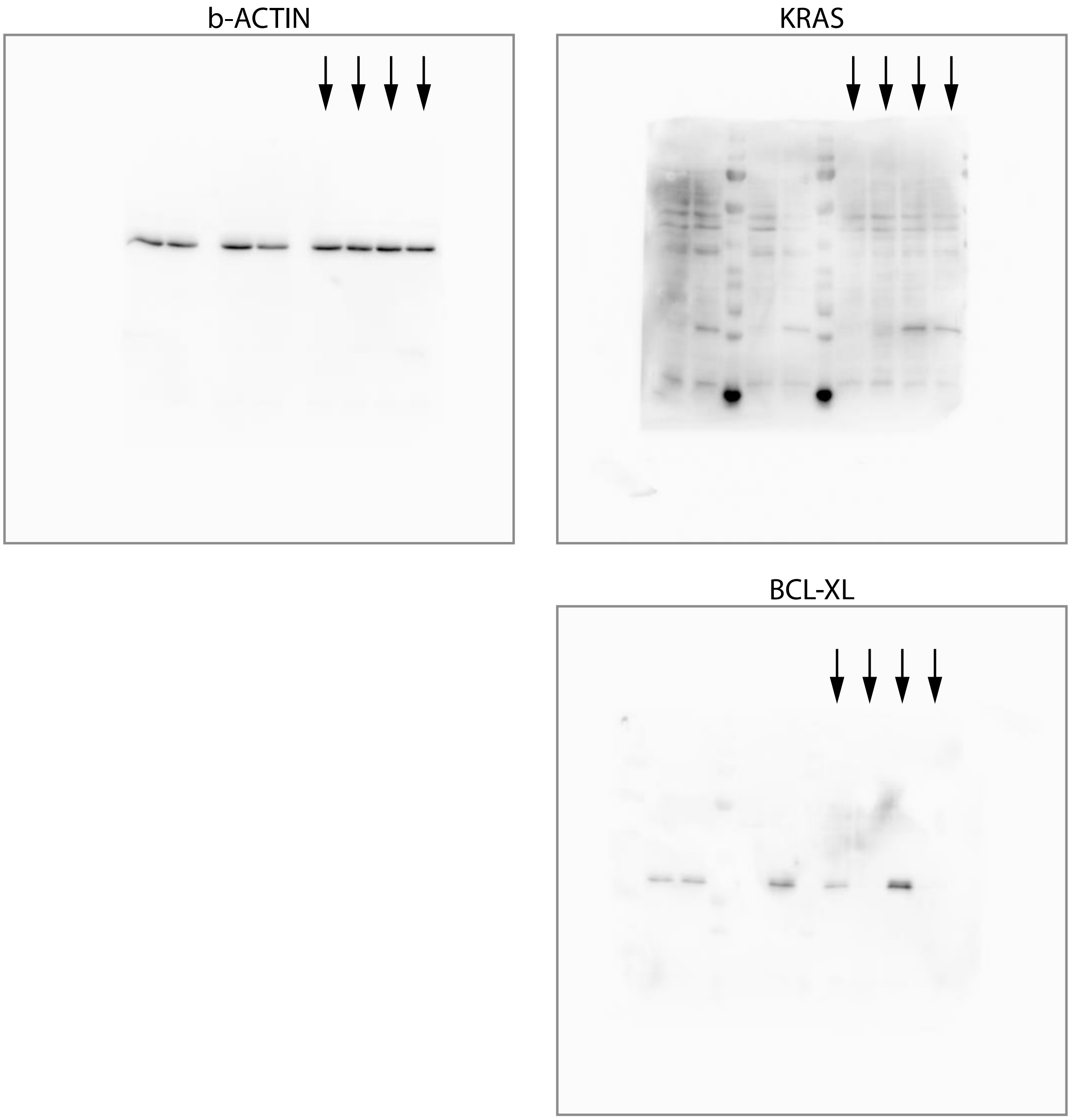

Supplementary Figure 8 | Full blots corresponding to portion of blots presented in Figure 3b
The lanes used in this study are indicated with arrows

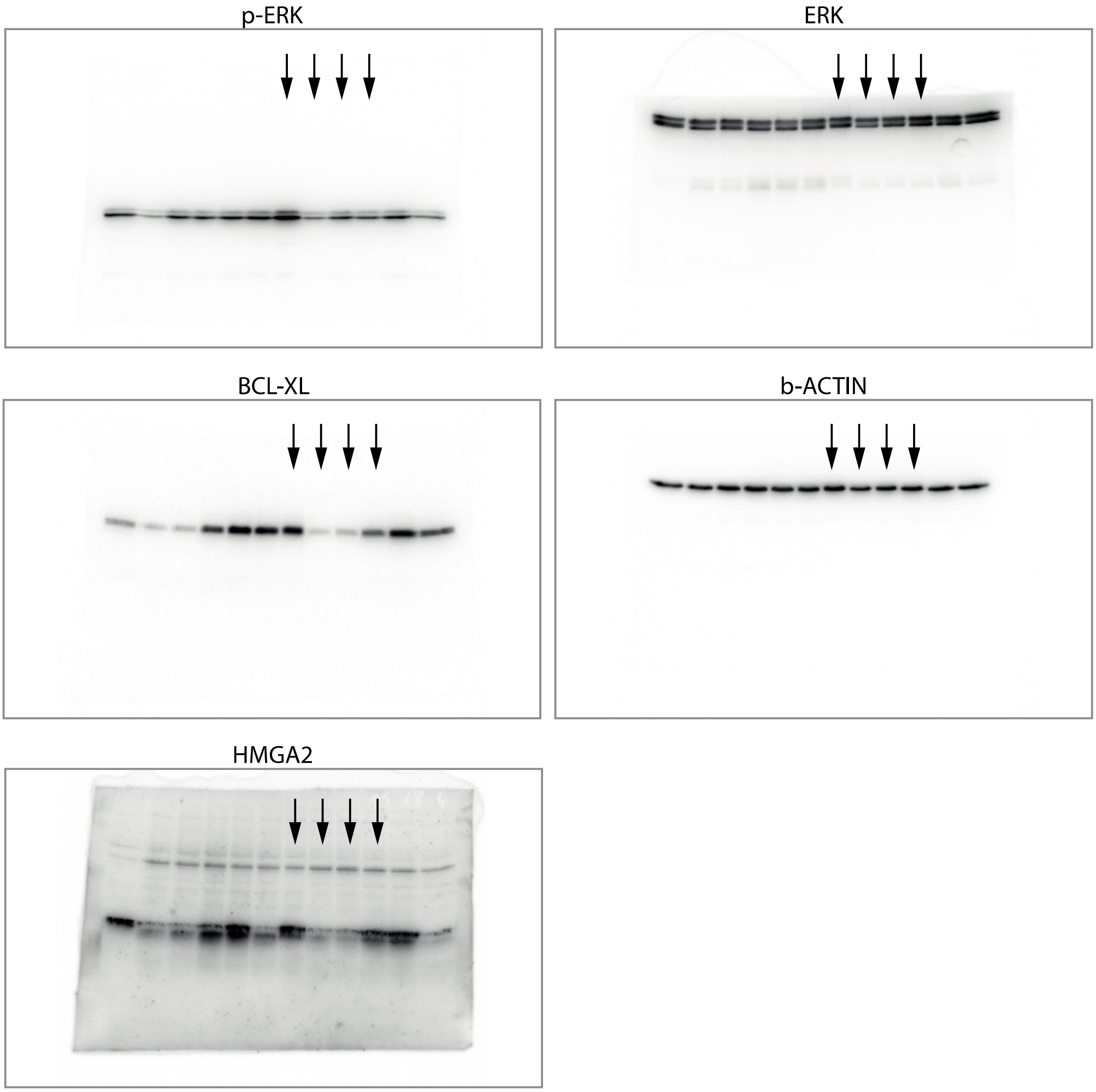

Supplementary Figure 8 | Full blots corresponding to portion of blots presented in Figure 3c, g

The lanes used in this study are indicated with arrows

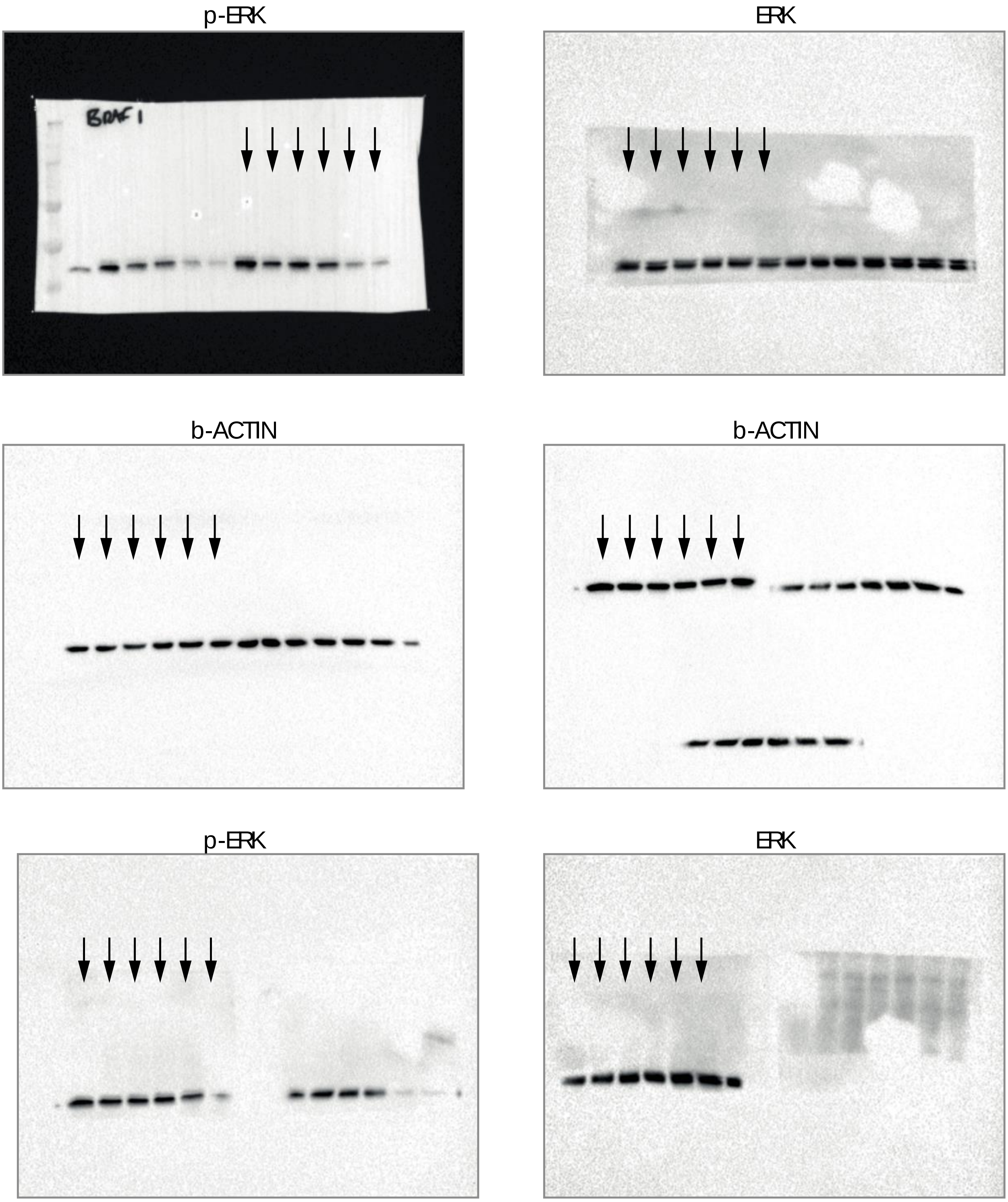

Supplementary Figure 8 | Full blots corresponding to portion of blots presented in Figure 4a, b

The lanes used in this study are indicated with arrows

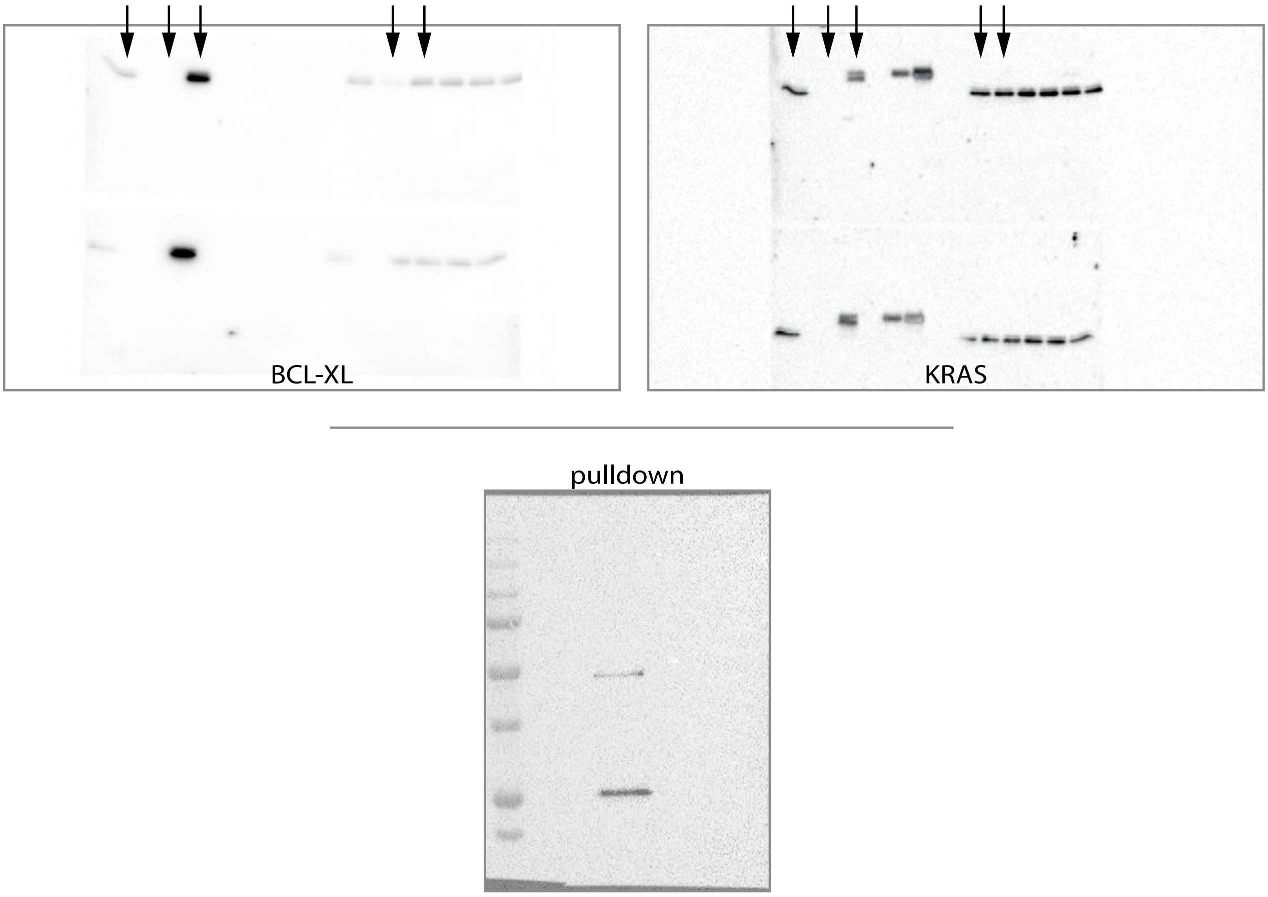

Supplementary Figure 8 | Full blots corresponding to portion of blots presented in Figure 5a
The lanes used in this study are indicated with arrows

SUPPLEMENTARY NOTE 1

MCF10A KRAS^{V12} cells are endowed with enhanced phenotypic plasticity, CIC properties and anti-apoptotic traits

Compared to the parental cells (MCF10A Lxsn) MCF10A KRAS^{V12} cells showed a typical mesenchymal morphology (Supplementary Figure 1e) and lost expression of epithelial markers to express mesenchymal markers as well as transcription factors involved in EMT (Supplementary Figure 1a). CIC traits include self-renewal and asymmetric division capacities that evoke those of stem cells and expression of cell surface markers (such as CD44 for breast CICs[1]). To evaluate the representation of CICs in bulk populations of MCF10A Lxsn and MCF10A KRAS^{V12} we measured the percentage of cells that self-renew and generate spheroid structures under low adherence and minimal culture conditions. Briefly, cells were seeded at very low concentration (to prevent cell-cell contact) in ultralow adherence plate in serum-free medium. Mammospheres were counted after 15 days incubation. Each cluster of more than 10 cells was considered as a mammosphere. The percentage of mammosphere-forming cells illustrates the percentage of cell featuring CIC properties from the bulk. MCF10A Lxsn were not able to grow under these conditions unless EGF was added to the culture medium to generate approximately 5% of mammosphere forming cells (Supplementary Figure 1b). In contrast, approximately 30% of KRAS^{V12}-transformed cells formed mammospheres (Supplementary Figure 1b). EGF-induced mammospheres were spherical and organised with a basal membrane whilst cells in KRAS^{V12}-induced mammospheres lost strong attachment and grew in unorganised clusters (Supplementary Figure 1f). MCF10A KRAS^{V12} cells harboured a population with significantly higher expression of CD44 compared to control cells (Supplementary Figure 1c). Young mammospheres derived from MCF10A KRAS^{V12} cells showed homogeneously high CD44 expression indicating that RAS-induced CICs are pre-existing in the CD44^{high} subpopulation (Supplementary Figure 1c).

Detachment from the extracellular matrix causes apoptosis of normal epithelial cells. This phenomenon is called anoïkis and oncogenic RAS has been shown to inhibits this[2, 3]. Ability to grow in sphere condition requires resistance to anoïkis, we thus asked if RAS activation in MCF10A cells conferred resistance to anoïkis. We evaluated anchorage-dependent cell death using methylcellulose in culture media to avoid cell-cell contact. We observed a decrease in cell death in KRAS^{V12} transformed cells compared to MCF10A Lxsn control cells indicating that KRASV12 conferred resistance to anoïkis (Supplementary Figure 1d). We also observed a decrease in apoptosis induced by staurosporine treatment in KRAS^{V12} transformed cells, further arguing that these cells are resistant to apoptosis induction (data not shown). Following this observation, we then asked if BCL-X$_L$ was responsible of CIC maintenance through the inhibition of apoptosis in this subset of cells.

SUPPLEMENTARY NOTE 2

BCL-X_L is not involved in CIC survival per se

To go further, we asked if the decrease of CICs percentage in BCL-X_L-knockdown MCF10A $KRAS^{V12}$ cells resulted from the loss of BCL-X_L anti-apoptotic activity in this subpopulation. We first evaluated whether growth in mammosphere forming conditions per se was accompanied with cell death in MCF10A $KRAS^{V12}$ cells. We did not detect cell death under these conditions, even after BCL-X_L depletion (Supplementary Figure 3a). We also quantified anoïkis in BCL-X_L-depleted cells. As shown in Supplementary Figure 1 $KRAS^{V12}$-transformed cells were significantly more resistant to anoïkis than control cells and this was not reverted by BCL-X_L knockdown. This indicates that enhancement of BCL-X_L expression is not sufficient to account for the resistance of KRAS transformed cells to anoïkis.

We also investigated the effects of a treatment with the pan-caspases inhibitor Q-VD-OPh on mammosphere formation in MCF10A $KRAS^{V12}$ cell line expressing BCL-X_L or not. Q-VD-OPh had no effects on mammosphere formation in either condition. This indicates that BCL-X_L-dependent mammosphere formation does not involve caspase inhibition (and by inference inhibition of apoptotic cell death) in MCF10A $KRAS^{V12}$ cells (Supplementary Figure 3b).

We then used ABT-737, a small molecule compound that interferes with BCL-X_L anti-apoptotic function by inhibiting its binding to pro-apoptotic BH3 domains. ABT-737 treatment significantly decreased clonogenicity of doxorubicin-treated $KRAS^{V12}$ cells (Supplementary Figure 3d) consistent with the notion that BCL-X_L contributes to the chemoresistance of MCF10A $KRAS^{V12}$ cells via its anti-apoptotic activity. In contrast, ABT-737 did not interfere with the ability of MCF10A $KRAS^{V12}$ cells to form mammospheres in presence of Q-VD-OPh or not. This suggests that BCL-X_L does not contribute to mammosphere formation via its canonical, pharmacologically tractable survival activity.

Finally, we performed BH3 profiling assays of MCF10A $KRAS^{V12}$ and control cells grown in adherent culture conditions or as young mammospheres (Supplementary Figure 3e). BH3 profiling is a functional analysis that uses peptides derived from the BH3 domains of pro-apoptotic BCL-2 family proteins to diagnose specific dependencies on BCL-2 family members in cell populations[4]. Cytochrome-c release was measured by flow cytometry following incubation with BH3 peptides of permeabilised cells[5]. Apoptotic priming in the bulk of MCF10A $KRAS^{V12}$ was lower than in control cells as judged by their dose-dependent response to the BAX/BAK activator peptide BIM-BH3. Cells from mammospheres showed a modest enhancement in priming that was not correlate with an enhanced dependency on BCL-X_L. Indeed RAS-activated cells grown in adherence or as mammospheres were neither sensitized to the BCL-2/BCL-X_L inhibitor peptide BAD-BH3 nor to the specific BCL-X_L inhibitor peptide HRK-BH3. They were instead more resistant than control cells to the latter peptide than they were to the MCL-1 inhibitor NOXA-BH3. EGF-treated cells and mammospheres-derived cells showed no enhancement of apoptotic priming and, a fortiori, no enhancement in BCL-X_L or MCL-1 dependency. As a whole, these data indicate that cells growing in conditions that favour mammosphere formation (RAS activation or EGF-treatment) and cells from mammospheres do not have an enhanced apoptotic load on BCL-X_L that would render this protein necessary for the survival of mammosphere forming cells.

SUPPLEMENTARY NOTE 3

Investigation of BCL-X_L interactions with KRAS using BRET assays

BRET assays evaluate the close proximity between two proteins within whole live cells by measuring energy transfer between R-Luciferase (R-Luc) fused to one protein and Yellow Fluorescent Protein (YFP) fused to the other upon addition of a Luciferase substrate. The saturability of signals observed between a given level of donor and increasing levels of acceptor together with the use of mutants as a negative control testify to specific interactions. We used such assays to investigate interactions between BCL-X_L and KRAS under different conditions in a whole cell context. Saturable BRET signals were observed between R-Luc fused wild type KRAS and YFP-fused BCL-X_L in HeLA cells, and interactions were confirmed by co-immunoprecipitation assays. These assays incidentally showed that YFP-BCL-X_L also interacts with endogenous wild type KRAS in these cells. BRET signals observed using a mutant R-Luc KRAS carrying the G12V substitution showed differences in the maximal BRET signal and in the BRET50. Further experiments are required to explore whether these differences reflect a genuine change in the stoichiometry and affinity of KRAS binding to BCL-X_L in whole cells. The saturability of the signal we observed in all cases indicates that KRAS interacts with BCL-X_L regardless of its mutational status. These assays were performed using the KRASA isoform but BRET signals measured using R-Luc KRASB showed that BCL-X_L also interacts with this isoform.

We also used the BRET technology to measure RAS activity in whole cells stimulated or not with EGF and the impact of overexpressed BCL-X_L (wild type or BH4 deleted) on this activity. To this end we used a BRET-based RAS activity sensor derived from Mochizuki et al., 2001 designed to measure interactions between R-Luc fused wild type KRAS and YFP-fused to the N-terminal end of the RAS binding domain of RAF (residues 49-132) in a whole cell context[6].

SUPPLEMENTARY METHODS

iTraq-MS

Extraction and digestion of proteins

Approximately 5x10^6 cells were lysed in 0.6 ml of 4% SDS and 0.1 M DTT in 0.1 M Tris-HCl, pH 7.6 at room temperature for 30 min and briefly sonicated to reduce viscosity of the lysate. Detergent was removed from the lysates and the proteins were digested with trypsin using the FASP protocol using spin ultrafiltration units of nominal molecular weight cut of 30 000. Briefly, the protein lysate was applied to an YM-30 microcon filter unit (Cat No. MRCF0R030, Millipore) spun down and washed three times with 200 µL of 8 M urea in 0.1 M Tris/HCl, pH 8.5. Then 6 µL of 200 mM MMTS in 8 M urea was added to the filters and the samples were incubated for 20 min. Filters were washed thrice with 200 µL of 8 M urea in 0.1 M Tris/HCl, pH 8.5, followed by six washes with 100 µL 0.5M TEAB. Finally, trypsin (AB sciex) was added in 100 µL 0.5M TEAB to each filter. The protein to enzyme ratio was 100:1. Samples were incubated overnight at 37°C and released peptides were collected by centrifugation. Samples were then dried completely using a Speed-Vac and re-suspended in 100 µl of 0.5% trifluoroacetic acid (TFA) in 5% acetonitrile, and desalted via PepClean C-18 spin columns (Pierce Biotechnology, Rockford, IL). Peptide content was determined using a Micro BCA Protein Assay Kit (Pierce-Thermo Scientific, Rockford, IL).

Peptide labeling with iTRAQ reagents

100 µg of each peptide solution was labeled at room temperature for 2h with one iTRAQ reagent vial previously reconstituted with 70µl of ethanol for 4plex iTRAQ reagent. Labeled peptides were subsequently mixed in a 1:1:1:1 ratio and dried completely using a Speed-Vac.

Peptide OFFGEL fractionation

For pI-based peptide separation, we used the 3100 OFFGEL Fractionator (Agilent Technologies, Böblingen, Germany) with a 12 or 24-well set-up using our protocol. Prior to electrofocusing, samples were desalted onto a Sep-Pak C18 cartridge (Waters). For the 24-well set-up, peptide samples were diluted to a final volume of 3.6 mL using OFFGEL peptide sample solution. To start, the IPG gel strip of 24 cm-long (GE Healthcare, München, Germany) with a 3-10 linear pH range was rehydrated with the Peptide IPG Strip Rehydradation Solution, according to the protocol of the manufacturer, for 15 min. Then 150 µL of sample was loaded in each well. Electrofocusing of the peptides was performed at 20°C and 50 µA until the 50 kVh level was reached. After focusing, the 24 peptide fractions were withdrawn and the wells were washed with 200 µL of a solution of water/methanol/formic acid (49/50/1). After 15 min, each washing solution was pooled with its corresponding peptide fraction. All fractions were evaporated by centrifugation under vacuum and maintained at -20°C. For the 2D-OFFGEL approach, the peptides were first fractionated in 12 fractions in the pH range 3-10. Then, fractions F1-F2, fraction F3 to F8 and fractions F9 to F12 were pooled and refractionated in 24 fractions in the pH range 3.5-4.5, 4-7 and 6-9, respectively. We obtained 72 fractions that were analysed by nanoLC-MS/MS.

Capillary LC separation

Just before nano-LC, the fractions were resuspended in 20 µL of H2O with 0.1% (v/v) TFA. The samples were separated on an Ultimate 3,000 nano-LC system (Dionex, Sunnyvale, USA) using a

C18 column (PepMap100, 3μm, 100A, 75μm id x 15cm, Dionex) at 300nL/min a flow rate. Buffer A was 2% ACN in water with 0.05% TFA and buffer B was 80% ACN in water with 0.04% TFA. Peptides were desalted for 3 min using only buffer A on the precolumn, followed by a separation for 105 min using the following gradient: 0 to 20% B in 10 min, 20% to 45% B in 85 min and 45% to 100% B in 10 min. Chromatograms were recorded at the wavelength of 214 nm. Peptide fractions were collected using a Probot microfraction collector (Dionex). We used CHCA (LaserBioLabs, Sophia-Antipolis, France) as MALDI matrix. The matrix (concentration of 2mg/mL in 70% ACN in water with 0.1% TFA) was continuously added to the column effluent via a micro "T" mixing piece at 1.2 μL/min flow rate. After 12 min run, a start signal was sent to the Probot to initiate fractionation. Fractions were collected for 10 secs and spotted on a MALDI sample plate (1,664 spots per plate, Applied Biosystems, Foster City, CA.).

MALDI-MS/MS

MS and MS/MS analyses of off-line spotted peptide samples were performed using the 5800 MALDI-TOF/TOF Analyser (ABsciex) and 4000 Series Explorer software, version 4.0. The instrument was operated in positive ion mode and externally calibrated using a mass calibration standard kit (ABsciex). The laser power was set between 2800 and 3400 for MS and between 3600 and 4200 for MS/MS acquisition. After screening all LC-MALDI sample positions in MS-positive reflector mode using 2000 laser shots, the fragmentation of automatically-selected precursors was performed at a collision energy of 1kV using air as collision gas (pressure of ~ 2 x 10-6 Torr) with an accumulation of 3000 shots for each spectrum. MS spectra were acquired between m/z 1000 and 4000. For internal calibration, we used the parent ion of Glu1-fibrinopeptide at m/z 1570.677 diluted in the matrix (30 femtomoles per spot). Up to 12 of the most intense ion signals per spot position having a S/N > 20 were selected as precursors for MS/MS acquisition. Peptide and protein identification were performed by the ProteinPilotTM Software V 4.0 (AB Sciex) using the Paragon algorithm as the search engine. Each MS/MS spectrum was searched for Homo sapiens species against the Uniprot/swissprot database (UniProtKB/Sprot 20110208 release 01, with 525997 sequence entries). The searches were run using the fixed modification of methylmethanethiosulfate labeled cysteine parameter enabled. Other parameters, such as tryptic cleavage specificity, precursor ion mass accuracy and fragment ion mass accuracy, are MALDI 5800 built-in functions of ProteinPilot software. The detected protein threshold (unused protscore (confidence)) in the software was set to 1.3 to achieve 95% confidence, and identified proteins were grouped by the ProGroup algorithm (ABsciex) to minimize redundancy. The bias correction option was executed.

A decoy database search strategy was also used to estimate the false discovery rate (FDR), defined as the percentage of decoy proteins identified against the total protein identification. The FDR was calculated by searching the spectral against the Uniprot Homo sapiens decoy database.

Quantification of relative protein expression

We employed a customized software package, iQuantitator, to infer the magnitude of change in protein expression. The software infers treatment-dependent changes in expression using Bayesian statistical methods. We used this approach to generate means, medians, and 95% credible intervals (upper and lower) for each treatment-dependent change in protein expression by using peptide-level

data for each component peptide. For proteins whose iTRAQ ratios were down-regulated, the extent of down-regulation was considered further if the higher limit of the credible interval had a value lower than 1. Conversely, for proteins whose iTRAQ ratios were up-regulated, the extent of up-regulation was considered further if the lower limit of the credible interval had a value greater than 1. The width of these credible intervals depends on the data available for a given protein. Since the number of peptides observed and the number of spectra used to quantify the change in expression for a given protein are taken into consideration, it is possible to detect small but significant changes in up- or down-regulation when many peptides are available. The peptide selection criteria for relative quantification were performed as follows. Only peptides unique for a given protein were considered for relative quantification, excluding those common to other isoforms or proteins of the same family. Proteins were identified on the basis of having at least two peptides with an ion score above 95% confidence.

BH3 Profiling

Experiments were performed as previously described [5, 7]. Briefly, cells were suspended in DTEB and added to peptides in DTEB/0.002% digitonin (w/v) for 60 min at 25 1C prior to fixation for 15 min with 2% formaldehyde. Formaldehyde was neutralized by the addition of 400mM glycine/570mM Tris-HCl pH 9.1 for 15 min. Staining for cytochrome-c (Clone 6H2.B4, BD Bioscience) was performed by addition of saponin, FBS Q12 and BSA to a final concentration of 0.1%, 2%, and 1% (w/v), respectively, followed by storage overnight at 4 °C prior to FACS analysis and evaluation of percentage of cytochrome-c release.

SUPPLEMENTARY REFERENCES

1. Al-Hajj M, Clarke MF. Self-renewal and solid tumor stem cells. *Oncogene* 23, 7274-7282 (2004).

2. McFall A, Ulku A, Lambert QT, Kusa A, Rogers-Graham K, Der CJ. Oncogenic Ras blocks anoikis by activation of a novel effector pathway independent of phosphatidylinositol 3-kinase. *Mol Cell Biol* 21, 5488-5499 (2001).

3. Liu Z*, et al.* Oncogenic Ras inhibits anoikis of intestinal epithelial cells by preventing the release of a mitochondrial pro-apoptotic protein Omi/HtrA2 into the cytoplasm. *J Biol Chem* 281, 14738-14747 (2006).

4. Ni Chonghaile T*, et al.* Pretreatment mitochondrial priming correlates with clinical response to cytotoxic chemotherapy. *Science* 334, 1129-1133 (2011).

5. Ryan J, Letai A. BH3 profiling in whole cells by fluorimeter or FACS. *Methods* 61, 156-164 (2013).

6. Mochizuki N*, et al.* Spatio-temporal images of growth-factor-induced activation of Ras and Rap1. *Nature* 411, 1065-1068 (2001).

7. Bah N*, et al.* Bcl-xL controls a switch between cell death modes during mitotic arrest. *Cell Death Dis* 5, e1291 (2014).
