# Supplementary material for: BCL-XL directly modulates RAS signalling to favour cancer cell stemness
Source: Nat Commun. 2017 Oct 24;8:1123. doi: 10.1038/s41467-017-01079-1 (PMC5654832; doi:10.1038/s41467-017-01079-1)
Supplement: Supplementary file 3 — Description of Additional Supplementary Files [file 41467_2017_1079_MOESM3_ESM.pdf]

Description of Additional Supplementary Files

File Name: Supplementary Data 1
Description: Ingenuity analysis of upstream regulators of RAS-induced BCL-XL-dependent target expression.

File Name: Supplementary Data 2
Description: A list of the proteins identified by mass spectrometry after IP-BCLXL in KRASV12-transformed cells only. Mass spectrometric analysis of protein lysates from KRASV12-transformed cells after IP-BCLXL was performed. BCL-XL itself was systematically identified in these immunoprecipitations. Negative control experiments were performed in parallel using lysates from the parental cells poorly expressing BCL-XL, with resulting immunoprecipitates harbouring no detectable BCL-XL. Proteins that were nevertheless identified in these negative control immunoprecipitates were classified as non-specific and they were taken off the short list of candidates shown in this table.
